# Cost-Effectiveness of Population Screening Programs for Cardiovascular Diseases and Diabetes in Low- and Middle-Income Countries: A Systematic Review

**DOI:** 10.3389/fpubh.2022.820750

**Published:** 2022-03-08

**Authors:** Manushi Sharma, Renu John, Sadia Afrin, Xinyi Zhang, Tengyi Wang, Maoyi Tian, Kirti Sundar Sahu, Robert Mash, Devarsetty Praveen, K. M. Saif-Ur-Rahman

**Affiliations:** ^1^The George Institute for Global Health, New Delhi, India; ^2^Health Systems and Population Studies Division, International Centre for Diarrhoeal Disease Research, Bangladesh (ICDDR, B), Dhaka, Bangladesh; ^3^The George Institute for Global Health at Peking University Health Science Center, Beijing, China; ^4^School of Public Health, Harbin Medical University, Harbin, China; ^5^Faculty of Medicine and Health, The George Institute for Global Health, University of New South Wales, Sydney, NSW, Australia; ^6^School of Public Health Sciences, University of Waterloo, Waterloo, ON, Canada; ^7^Department of Family and Emergency Medicine, Stellenbosch University, Stellenbosch, South Africa; ^8^Faculty of Medicine and Health, University of New South Wales, Sydney, NSW, Australia; ^9^Prasanna School of Public Health, Manipal Academy of Higher Education, Manipal, India; ^10^Department of Public Health and Health Systems, Graduate School of Medicine, Nagoya University, Nagoya, Japan

**Keywords:** evidence synthesis, non-communicable chronic diseases prevention, economic evaluation, prevention, primary health care (PHC), health technology assessment (HTA)

## Abstract

Almost all low- and middle-income countries (LMICs) have instated a program to control and manage non-communicable diseases (NCDs). Population screening is an integral component of this strategy and requires a substantial chunk of investment. Therefore, testing the screening program for economic along with clinical effectiveness is essential. There is significant proof of the benefits of incorporating economic evidence in health decision-making globally, although evidence from LMICs in NCD prevention is scanty. This systematic review aims to consolidate and synthesize economic evidence of screening programs for cardiovascular diseases (CVD) and diabetes from LMICs. The study protocol is registered on PROSPERO (CRD42021275806). The review includes articles from English and Chinese languages. An initial search retrieved a total of 2,644 potentially relevant publications. Finally, 15 articles (13 English and 2 Chinese reports) were included and scrutinized in detail. We found 6 economic evaluations of interventions targeting cardiovascular diseases, 5 evaluations of diabetes interventions, and 4 were combined interventions, i.e., screening of diabetes and cardiovascular diseases. The study showcases numerous innovative screening programs that have been piloted, such as using mobile technology for screening, integrating non-communicable disease screening with existing communicable disease screening programs, and using community health workers for screening. Our review reveals that context is of utmost importance while considering any intervention, i.e., depending on the available resources, cost-effectiveness may vary—screening programs can be made universal or targeted just for the high-risk population.

## Introduction

Cardiovascular diseases (CVDs) and diabetes are the non-communicable diseases (NCDs) that contribute the most to the increasing burden of disease and add to the challenges of health systems in low- and middle-income countries (LMICs) (1). Early detection of CVD and diabetes can alleviate the burden on health systems, particularly the financial consequences (2, 3). The most effective way of managing this onslaught of NCDs is pairing curative services with strategically planned and tailored preventive interventions, such as screening, especially in the context of attaining and sustaining Universal Health Coverage (UHC) (4–6). The global trend toward more screening and case finding for NCDs has increased during the past decade (7). However, in many cases, a clear evidence base for the cost-effectiveness of screening is missing (8–10). Policymakers, managers, and other stakeholders are vulnerable to the “prevention is better than cure” mantra and tend to generalize evidence without appraising its feasibility uncritically and can cause inadvertent financial harm to their health systems (10). Affordability and feasibility are crucial pieces of evidence in decision making, along with clinical evidence of effectiveness, epidemiological factors, and the social and cultural context (11, 12). Traditionally, economic evaluations are performed for “budget burners” or high-cost interventions such as cancer drugs or clinical treatments for NCDs (13). These evaluations are equally crucial for preventive interventions, mainly when adopted for whole populations (14, 15). In a brief review of existing literature, NCD interventions like screening, when not tailored to the populations' characteristics, have the potential of causing more harm to the health system than good (15, 16). There is a scarcity of economic evaluations in LMICs, especially for NCD prevention (10, 15). This systematic review aims to consolidate and synthesize evidence of the costs and benefits of screening interventions for diabetes and CVDs in LMICs. Specifically, our objectives are to review the economic evaluations of screening programs for early detection of CVD and diabetes; and identify the gaps in evidence of the cost-effectiveness of screening programs from LMICs to provide direction for future research.

## Methods

We used the Preferred Reporting Items for Systematic reviews and Meta-analyses (PRISMA) (17) approach for reporting this study. The protocol was registered on PROSPERO (CRD42021275806).

### Eligibility Criteria and Information Sources

The inclusion and exclusion criteria as summarized in Box 1. Our interest was in population or mass screening strategies that aimed at early detection of CVD and diabetes. We adapted the WHO definition of screening: presumptive identification of unrecognized disease or defects through tests, examinations, or other procedures applied rapidly in a population (18).

Box 1 **Inclusion and exclusion criteria.**

**Inclusion**

- Types of Studies: Full or partial economic evaluations (cost effectiveness analysis, cost utility analysis, cost benefit analysis, cost minimization analysis), model based, or trial based.
- Population: All population in LMICs
- Intervention: Population screening and screening interventions paired with clinical management strategies
- Outcomes of interest: costs and cost-effectiveness as incremental cost per quality-adjusted life years (QALYs) gained, or disability-adjusted life years (DALYs) averted, or life years gained or intermediate outcomes
- Time: Articles published in the past 10 years (From 1st August 2011 till 31st July 2021)
- Language: studies published in English and Chinese (Mandarin).

**Exclusion**

- Economic evaluations related to management of complications of diabetes and cardiovascular diseases
- Reviews, commentaries (letters to the editors, editorials), congress abstracts.

We included studies in English and Chinese (Mandarin) because of the review teams' experience and proficiency in both these languages. Secondly, our aim was to be thorough in review of literature from LMICs. China qualifies and is one of the few countries with an academic database We searched Medline through PubMed, Scopus, Embase, Econlit, Web of Science, Cost-Effectiveness Analysis Registry, China National Knowledge Infrastructure (CNKI), Wanfang Data Knowledge Service Platform (Wanfang), and China Science and Technology Journal Database (Weipu) from 1st August 2011 until 31st July 2021. The past 10 years have seen a rise economic evaluations of preventive health interventions and policies in LMICs and thus a 10-year time frame was chosen for this study. This search was supplemented by a manual search of the references and expert consultation. The search strategy is described below.

### Search Strategy

Our search strategy and keyword identification follow the peer review of the electronic search strategies (PRESS) checklist (19). In the search strategy, medical subject heading (MeSH) terms and relevant keywords related to cost-effectiveness, screening, diabetes, cardiovascular diseases, and prevention were used to cover all articles on the topics. The review team searched for articles in English and Chinese (Mandarin). The electronic search strategy used for all the databases is in Annexure I.

### Review Process

Records were imported into Ryyan software for de-duplication and screening. Four independent authors, two (MS and SA) for English and two (XZ and TW) for Chinese, reviewed all titles and abstracts, while a third reviewer resolved disagreements. Advice from other co-authors was sought where necessary. Finally, full-text screening was conducted independently against the eligibility criteria by MS and RJ for English and XZ and TW for Chinese.

#### Critical Appraisal of Included Studies

The Joanna Briggs Institute's (JBI) Critical Appraisal tool (20) for the quality assessment of economic evaluation studies was used to assess the methodological quality of the included studies. The JBI's checklist is the tool of choice because it is designed specifically for assessing the quality of economic evaluations compared to other tools such as CHEERS which provide guidance on reporting of economic evaluation. Each of the included studies was evaluated against 11 questions, and the quality score was calculated by adding up all the points for the questions answered with “yes.” The maximum possible score is eleven, indicating high quality. Two reviewers independently completed the tool from the English and Chinese teams, and each study was assigned a score. Disagreements were resolved by group discussions and consensus amongst the co-authors.

### Data Analysis, Interpretation, and Reporting

Four review authors (MS and SA for English, and XZ and TW for Chinese) extracted and collated data from the studies to summarize key characteristics such as setting, intervention, type of economic evaluation, cost-effectiveness threshold, discount rate, time horizon, study perspective, methods, outcomes reported, and outcome values. The definitions of key concepts of economic evaluations are listed below:

- The **study perspective** is the point of view adopted by the researchers when deciding on which types of costs and health benefits should be included in the economic evaluation (21).
- **Costs** used in an economic evaluation are calculated as a product of counts of items of resource use associated with a patient's care and a standard “unit” cost of each type of item (22). Estimates are derived from different survey and registry sources, converted to costs using representative “unit costs,” and then aggregated across relevant population cohorts (23).
- The **time horizon** used for an economic evaluation is the duration over which health outcomes and costs were calculated (24).
- The **cost-effectiveness threshold** is the maximum amount a decision-maker is willing to pay for a unit of health outcome (25).
- Costs and health **outcomes** predicted to occur in the future are usually valued less than present costs, so they are discounted in analysis. Therefore, the results are expressed as a series (streams) of health outcomes and costs over time, applying a discounting factor to each value in the series and then aggregating to give each stream a “present value” (26).
- In economic evaluation of healthcare interventions, **utilities** or health state preference values represent the strength of individuals' preferences for different health states. When these are averaged over a population, the result is valuations of health states (27).
- **Sensitivity analysis** illustrates and assesses the level of confidence that may be associated with the conclusion of an economic evaluation. It is performed by varying key assumptions made in the assessment and recording the impact on the result (output). The values of key input parameters may vary, and structural assumptions on how the parameters are combined in the model. Sensitivity analysis can be one-way, where input parameters are run one by one; multi-way, where more than one parameter is varied at the same time; “threshold” analysis where the model is used to assess the tipping point for an input parameter, or probabilistic—a stochastic approach to producing a distribution of outputs, based on the distribution of input parameters (28).
- **Budget impact analyses** estimate the likely change in expenditure for the payer for the choice that is made. A cost-effectiveness analysis forecasts a value for money, and studies using budget impact models assess affordability (29).

The extracted data is summarized in a narrative using descriptive statistics. A meta-analysis was not possible given the heterogeneity in the studies and population characteristics.

## Results

### Search Results

**Figure 1:**
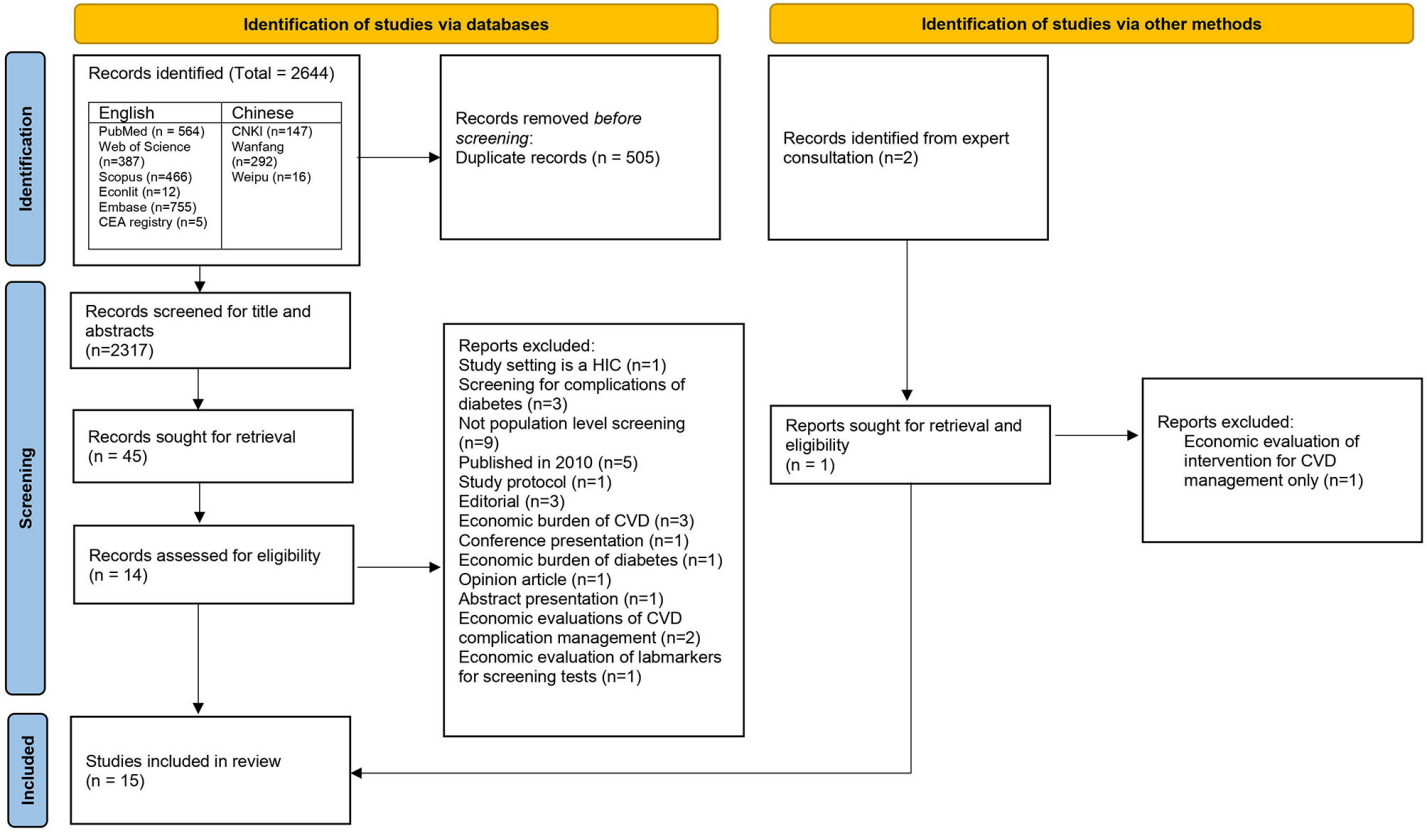
PRISMA diagram.

English Studies: An initial search retrieved 2,189 potentially relevant publications from all the databases. Out of these 2,189 publications, there were 849 duplicates, representing 326 unique articles. Therefore, the final number of unique articles screened for titles and abstracts were 1,683. Articles that were economic evaluations of cancer screening strategies, economic or epidemiological burden of diseases, studies on health financing of NCDs, systematics reviews, editorials and opinion pieces were excluded. Finally, 36 articles moved to the next step of full-text screening. In this step, 12 articles were selected for final review. Causes of exclusion are listed in the Figure 1. In addition, one further article was included after reference search, gray literature search, and consultation from an expert in primary health care. Finally, a total of 13 articles identified from the English databases were included in the final review.

Chinese studies: We identified 455 articles from the initial search. After the title and abstract screening, a total of 2 articles met the inclusion criteria and were selected for the final review.

Out of the **total 15 articles**, five studies, whilst purporting to be full-fledged cost-effectiveness analysis, were costing analysis (30), cost-minimization analysis (31–33), and cost-benefit analysis (5). A flow of selection process as per the PRISMA guidelines is in Figure 1.

### Settings and Population

The identified articles represented a wide range of LMICs: nine from South and East Asia (Bangladesh, Pakistan, Sri Lanka, Vietnam, Indonesia, Bhutan, China, Thailand) (5, 16, 32–38), three from sub-Saharan Africa (South Africa, Nigeria, Uganda) (30, 39, 40), one multi-country study (South Africa, Mexico, and Guatemala) (41), one from Latin America (Brazil) (42), and one from Europe (Serbia) (31).

All the studies evaluated interventions provided through primary care (Supplementary Table 1)—five articles targeted rural populations, seven articles evaluated interventions for national populations, and one article targeted military personnel. The average ages targeted by screening programs were all over 40 years of age.

### Types of Intervention

The screening interventions were divided into three main categories (Supplementary Table 1): six studies described interventions focusing on CVD (31, 34, 36, 38, 39, 41), five studies described interventions focusing on diabetes (5, 32, 33, 37, 42), and four studies described interventions focusing on both CVD and diabetes (16, 30, 35, 40).

### Types of Economic Evaluation

Of the total fifteen studies, nine studies were cost-effectiveness analyses (CEA) (16, 34, 36–42), one was a cost-benefit analysis (CBA) (5), one was a cost-utility analysis (CUA) (35), three were cost-minimization analyses (CMA) (31–33), and one was an incremental cost analysis (30).

#### Study Perspective and Costs

Of the total studies, 11 adopted a health payer perspective (30–34, 36, 38–42), four studied costs from a societal perspective (5, 16, 35, 37). A summary of different costs included in each study is given in Supplementary Table 1.

#### Time Horizon

Amongst the included studies, five articles analyzed outcomes over a lifetime (16, 35, 36, 41, 42), four took a ten-year time frame (34, 38–40), one took a 40-year time frame (37), and five studies did not mention a time frame (5, 30–33).

#### Threshold

Out of the 15 records, five studies used a gross domestic product (GDP) based threshold. Out of these five studies, four used three times the GDP per capita (35, 36, 41, 42), and one used one-and-a-half times the GDP per capita (30). Five other studies adopted a willingness to pay threshold (16, 34, 37–39), while six did not use any threshold.

#### Study Design

Among the included articles, four studies employed a Markov model (16, 37–39, 42). The Brazilian study adopted the Center of Disease Control (CDC)/Research Triangle Institute (RTI)' cost-effectiveness model for type 2 diabetes (T2DM). A validated Markov model was populated with data from the Brazilian national population-based screening program (42). Other studies developed purpose-built Markov models (16, 37–39). The researchers in Uganda developed a cost epidemiologic model to study the health impact, costs, and cost-effectiveness of integrating basic screening and treatment services for hypertension, diabetes mellitus, and hypercholesterolemia in human immunodeficiency virus (HIV) treatment services (40). A cluster-randomized controlled trial was conducted in 30 rural communities in Bangladesh, Pakistan, and Sri Lanka, and costs were quantified prospectively (36). The Serbian researchers performed a retrospective cost preventive study (31). The researchers from South Africa conducted a cross-sectional costing analysis of an integrated HIV-NCD home-based testing and counseling program (30). The Thai researchers analyzed one-time screening performance and costs in a cost-benefit analysis (5). The two Chinese studies were CMAs that compared the average cost of different screening strategies (32, 33, 43).

#### Discount Rate and Utility Scores

Nine out of 15 studies used a 3% discount rate (16, 30, 34–38, 41, 42), while other studies did not specify a discount rate.

Amongst the included articles, seven articles did not mention the tools employed for calculating utility explicitly, they derived the values from WHO life tables or the global burden of disease databases. As such, upon review of a methodological article (44) we grouped them under health utility index (16, 34–36, 39, 40, 42), followed by two articles used the EQ-5D index scores (37, 41), one study used the SF-6D index score (38) and the remaining four studies did not mention utility scores (5, 31–33).

#### Sensitivity Analysis

The summary of the studies and types of sensitivity analysis is in Supplementary Table 1. Notably, the sensitivity analysis revealed that the COBRA-BPS (36) would also remain cost-effective if, all else being equal, mean incremental reductions of systolic blood pressure were no lower than 2.96 mm Hg in Bangladesh, 2.54 mm Hg in Pakistan, and 2.06 mm Hg in Sri Lanka, or if the percentage improvement in DALYs for each 1 unit decrease in systolic blood pressure remained above 1.48% in Bangladesh, 1.12% in Pakistan, and 0.73% in Sri Lanka. Paper-based and mobile-based strategies were sensitive to the costs of the statins in all three countries South Africa, Mexico, and Guatemala (41). In Vietnam (38), the results seemed relatively insensitive to changing values of input parameters in the 10-year model. In the lifetime model, the cost per quality-adjusted life year (QALY) was much lower after changing the high blood pressure prevalence and higher in a 3% utility discount scenario than the base case. In rural Nigeria (39), the cost-effectiveness was sensitive to variations in the discount rate, the effect of treatment on systolic blood pressure, delivery costs, inclusion of disability weights for being on antihypertensive treatment and costs of hypertension treatment. In the SMARThealth intervention (34), the results were most sensitive to the number of additional primary care visits by those identified as high risk receiving the intervention, the effectiveness of the intervention, and the proportion of the baseline population at increased risk of CVD. The intervention was most cost-effective when a higher proportion of the population was at high-CVD risk. In Uganda (40), integrating NCD screening and treatment with existing HIV infrastructure would be more cost-effective (net cost per disability-adjusted life year (DALY) averted decreased) with higher treatment effectiveness estimates and higher cost estimates for CVD treatment. While in South Africa screening efficiency was a key driver of program costs (30).

#### Budget Impact Analysis

Out of the fifteen records, only two studies performed a budget impact analysis (16, 36).

#### Outcomes Reported and Results

The outcomes and the results reported in each study are summarized in Supplementary Table 1. Amongst interventions targeting cardiovascular diseases, usage of mobile applications by community health workers proved to be more cost-effective than the paper-based option in South Africa, Mexico, and Guatemala. Incremental cost-effectiveness ratio (ICER) reported in Guatemala and Mexico was USD565 per QALY gained USD3.57 per QALY gained, and the use of the mobile application was cost-saving (it increased QALYs and reduced overall costs) in South Africa (41). Screening males from 55 years onwards was cost-saving for Vietnam's one-off, biannual and annual screening options with an ICER of Int$ 2,076 QALY gained (38). In Nigeria, screening, and treatment for hypertension under the national insurance program was potentially cost-effective. Still, the results were sensitive to changes in underlying assumptions with a wide range of uncertainty (39). The usage of SMARThealth mobile technology in Indonesia to screen and manage cardiovascular diseases was cost-effective compared to no SMARThealth gaining USD429 per DALY averted (34). The Serbian study calculated the average costs of screening active-duty military personnel for ischemic heart disease. The average costs of all services during the periodic-health-examination screening program were Euro 76.96 per subject. However, the average costs of all services during the periodic-health-examination screening program for patients with newfound arterial hypertension and poorly regulated arterial hypertension were Euro 767.54 per patient and Euro 2,103.63 per patient (31).

Amongst the studies that targeted diabetes—One study simulated four diabetes prevention strategies with a control group that had no prevention: (1) One-off screening for undiagnosed diabetes and impaired glucose tolerance (IGT), with lifestyle interventions on diet, (2) Only exercise and screening, (3) Only diet combined exercise (duo-intervention) in those with IGT and screening and (4) One-off screening alone without any lifestyle intervention (37). Independent age-specific models were simulated based on various incidences of diabetes, mortalities, and health utilities. They found that screening and promotion of exercise had the most significant savings at all three starting ages (37). In Brazil, the national diabetes screening program will yield considerable health benefits and higher costs. Compared with no screening, screen detection of undiagnosed diabetes resulted in USD 31,147 per QALY gained. In the base case analysis, not considering the intangible benefit of transferring diabetes management to primary care nor the benefit of using statin to treat eligible diabetic patients, cost-effectiveness ratios were not cost-effective considering thresholds proposed by the WHO (42). In the Thai study, CBA of different approaches to diabetes screening all screening methods using questionnaires were relatively more cost-effective than those using fasting plasma glucose. Their relative cost-effectiveness was, however, not obviously different (5). Another two studies identified the average costs of screening programs in China, recommending that screening be applied to only the high-risk populations (32, 33).

Four studies evaluated combined interventions for CVDs and diabetes. Two studies from Indonesia (16)—a CEA, and Bhutan (35)—a CUA were economic evaluations of the WHO's package of essential non-communicable (PEN) disease interventions for primary health care. Implementing the PEN program in Indonesia was better than no screening. However, it could be improved by targeting high-risk groups aged 40-years and above instead of screening 15-years and above as is the current practice with an average lifetime cost of is IDR57.66 million The current screening option in Bhutan, where overweight, obese, or those 40 years and older who visited primary care facilities were screened for diabetes and hypertension, represented good value for money compared to “no screening.” Also, expanding opportunistic screening (70% coverage of the target population) to universal screening (where 100% of the target population are screened) is likely to be even more cost-effective. The Ugandan study revealed that providing services for hypertension, diabetes, and high cholesterol for Ugandan antiretroviral therapy patients would reduce the overall CVD risk. It would amount to about 2.4% of national HIV/AIDS expenditure and would present cost-effectiveness comparable to other standalone interventions to address NCDs ranging from USD8,800 to USD1,400 per DALY averted, depending on the age and sex category (40). Integrated HIV-NCD screening in South Africa has the potential to utilize resources compared with standalone services efficiently. While all-inclusive NCD screening could increase the incremental cost per person screened for integrated HIV-NCD services USD3.95 (42%) per person screened (from USD9.36 to USD13.31 per person), a less costly lipid assay or targeted screening would result in a modest increase in costs with the potential to avert NCD death and disability (30).

### Quality Assessment

The overall quality of studies based on key methodological issues and the quality of evidence in each of the articles, including any potential for bias, are reported in Supplementary Table 2. In general, there was transparency in reporting of study questions and methodology. Detailed description and justification for measures used for costs and outcomes were provided. Four studies (5, 31–33) did not conduct a sensitivity analysis to establish the validity of the results or discuss the generalizability of the results. Most of the studies undertook an incremental cost analysis that reported the marginal shift in resources from the comparator to intervention. Critical appraisal of the studies revealed that nine had an overall score of 11 (maximum possible score) indicating high quality, three studies scored ten, one study scored nine, one recorded eight, and two studies scored four. All the studies reported the results of the economic evaluation comprehensively.

## Discussion

This review reveals a variety of screening interventions provided through primary care that are cost-effective in an LMIC context. Geographically, the studies were widespread—**seven** studies from South and East Asia out of which five are CEAs (34, 36–38, 42), one is a CBAs (5), one is a CUA (35), and two CMAs (32, 33). **Three** studies are from sub-Saharan Africa, out of which two are CEAs (39, 40) and one is an incremental cost analysis (30). **One** CEA from Latin America; **one** CEA—multi-country study set in Latin America and sub-Saharan Africa; and **one** cost analysis from Europe (31). The interventions targeted CVD screening, or diabetes screening, or both combined.

There is an increasing appetite for setting priorities in health care using real-time evidence through health technology assessments. Still, there is a lack of their application in preventive health program evaluation. This and one other review underscore the need for more high-quality economic evidence on such population screening programs (15). Although the evidence broadly supports the cost-effectiveness of screening programmes, more specific evidence is needed for approaches to population screening in different contexts. Though most of the studies used sensitivity analysis to investigate uncertainties in estimates of cost or outcome, there was limited use of this method to explore the implications of variability within and between different settings. Amongst the three studies identified from China, two were CMAs comparing average costs of different intervention strategies (32, 33) and do not provide a holistic picture for decision-making. There is a need to conduct economic evaluations that incorporate economic and epidemiologic factors to tailor interventions to the populations' needs. To ensure economic evaluations meet quality standards, LMICs should collaborate and create national or regional guidelines (45–47). Formal process and methods guidelines with utility scores, thresholds, and other contextual details would improve the quality of research and, to some extent, solve the problem of transferability of results (47).

Population screening is an integral part of NCD management strategy. Although, a Cochrane review concluded that population screening programs without follow-up are unlikely to be beneficial (48). A WHO report summarized current evidence indicating that screening for CVD risk factors does not reduce the CVD burden (49). Only two (34, 36) studies evaluated interventions that included screening and management of CVDs. Although, some studies did mention that screening programs were a part of clinical management strategies aimed at decreasing the burden of CVD and diabetes. Implementation depends on several factors—epidemiological, resources for further diagnosis and treatment, protocols for clinical management, sensitivity and specificity of the test, and cost-effectiveness (50). In this review, we focused only on the cost-effectiveness of screening programs. Another critical consideration is the capability and capacity of primary care and primary care providers to cope with a flood of new patients.

The review reveals a variety of innovative screening and management interventions designed and implemented in response to the rising burden of chronic diseases in LMICs. Innovation varied from integrating NCD screening into existing HIV screening (30, 40), using mobile health technology (34, 36), to employing and building the capacity of community health workers to screen, track and manage NCD in rural settings (30, 36, 41). All these pilots proved successful, thus calling policymakers to move beyond pilot testing to population-based screening approaches.

Countries and researchers continue to use a one-size-fits-all prescription despite wide criticism and ample evidence that uncritical adoption of international recommendations and guidelines may cause more harm than good (9, 10, 13, 43, 47, 48). Most studies used the WHO-CHOICE recommended 1 to 3 times GDP per capita threshold to ascertain cost-effectiveness. Thresholds should reflect the local health constraints; for instance, what may be cost-effective in the United Kingdom [public health expenditure as of 2020 is estimated at USD360 billion (51)] will be cost-ineffective in Indonesia [public health expenditure as of 2020 was ~USD15 billion (52)]. Very few countries have tailored thresholds. The WHO CHOICE threshold is too high for some countries, and in such cases, interventions will falsely prove to be cost-effective (10, 53–55). None of the studies justified using the 1 to 3 times GDP per capita as a threshold. Tailoring international guidelines to suit the country context and budget is of utmost importance (56). The Indonesian adoption of the PEN program is an example of how a “best buy,” when not tailored to the context, becomes a low value for money intervention, draining resources (16). Studies from Vietnam and China also substantiate the claim that screening a high-risk population is most cost-effective (37, 38). Thus, international guidelines should be scrutinized for transferability and feasibility before implementation.

The choice of the comparator is one of the main factors that influence the results of an economic evaluation. Modeling studies from Vietnam and China (37, 38) evaluated the cost-effectiveness ratio against “no screening.” A review of 29 pharmacoeconomic guidelines (57) concluded that the most recommended comparator (in 86% of the guidelines) is “the standard of care for local practices.” In choosing “no screening,” it is possible the comparator lies outside the efficiency frontier on the cost-effectiveness plane, biasing the analysis results.

Most of the studies were conducted on donor-based funding grants (which is the mainstay of health systems in most LMICs) (16, 34–36, 38, 39, 41). With a decline in donor funding due to the global recession (58, 59), governments should earmark funds for research in priority setting in health, including preventive programs such as screening to guide investments and ensure the sustainability of universal health coverage programs. There is also a need for strategic investments to build the capacity of both users (policymakers and high-level managers) and suppliers (academics, research organizations) of health technology assessments to ensure the agenda for priority setting is locally driven (45, 46, 60, 61).

The main strength of our study was the use of robust search and review methods. The search was not restricted to the English language but also included studies from Chinese databases; however, there was a lack of quality economic evidence of population screening strategies. Due to lack of knowledge of other languages we were restricted to only English and Chinese, which is also a limitation. We could not conduct a meta-analysis given the heterogeneity in the outcomes. The likelihood of missing important studies is very less. However, we did find an economic evaluation of population screening program for CVD and diabetes from India, which could not be included due to its publication date beyond the inclusion criteria of this study (54). Regardless, this study contributes to critical evidence on the cost-effectiveness of NCD screening programs in LMICs; it also gives an overview of the contextual population-level strategies employed to screen populations. We also detail the factors that contribute to cost-effectiveness in each setting.

## Conclusion

While the data is heterogenous it is evident that the success of a screening program depends upon context—epidemiological and social factors, political priorities, and budgetary constraints to name a few. Different permutations and combinations work for different contexts for, e.g., following screening with a management program, adopting a targeted approach rather than a universal approach. Our conclusions resonate with Eriksen et al. (49) screening alone is insufficient to improve health outcomes. Cost-effectiveness is an essential piece in the puzzle that needs to be judged in the local setting regarding the health system's available financial resources and capacity. Our review reveals that more economic evaluations of preventive programs for NCDs are required at national and regional levels to guide policymakers. We also identified shortcomings in the methodology to guide future research.

## Data Availability Statement

The original contributions presented in the study are included in the article/Supplementary Material, further inquiries can be directed to the corresponding author/s.

## Author Contributions

MS conceptualized and designed the research, analyzed data, interpreted results, and drafted the manuscript. KS-U-R, KS, and DP provided guidance and clarified any doubts. KS, RM, DP, and KS-U-R made a critical revision of the manuscript. RJ and SA screened English studies, extracted data, and performed the critical appraisal using the JBI Checklist. XZ and TW led the search, extraction, and analysis of the Chinese studies with guidance from MT. All authors contributed to writing, reviewing, and editing the manuscript and approved the submitted version.

## Funding

This study was conducted by the members of the Primary Health Care Research Consortium, which was funded by a grant from the Bill and Melinda Gates Foundation (INV-000970).

## Conflict of Interest

The authors declare that the research was conducted in the absence of any commercial or financial relationships that could be construed as a potential conflict of interest.

## Publisher's Note

All claims expressed in this article are solely those of the authors and do not necessarily represent those of their affiliated organizations, or those of the publisher, the editors and the reviewers. Any product that may be evaluated in this article, or claim that may be made by its manufacturer, is not guaranteed or endorsed by the publisher.

## Supplementary Material

Annexure 1 Search strategy.

Supplementary Table 1 Summary.

Supplementary Table 2 Quality assessment.
