# Supplementary material for: Cost-Effectiveness of Population Screening Programs for Cardiovascular Diseases and Diabetes in Low- and Middle-Income Countries: A Systematic Review
Source: Front Public Health. 2022 Mar 8;10:820750. doi: 10.3389/fpubh.2022.820750 (PMC8957212; doi:10.3389/fpubh.2022.820750)
Supplement: Annexure 1 — Search strategy. [file Data_Sheet_1.docx]

Cost and cost-effectiveness of population screening programs for cardiovascular diseases and diabetes in low- and middle-income countries - Evidence from a systematic review

**Search strategy: PubMed/Medline**

1. economic* OR price OR pricing OR expenditure* OR “value for money” OR budget* OR “return on investment” OR “value proposition” OR “Analyses, Cost-Benefit” OR “Analysis, Cost-Benefit” OR “Cost-Benefit Analyses” OR “Cost Benefit Analysis” OR “Analyses, Cost Benefit” OR “Analysis, Cost Benefit” OR “Cost Benefit Analyses” OR “Cost Effectiveness” OR “Effectiveness, Cost” OR “Cost-Benefit Data” OR “Cost Benefit Data” OR “Data, Cost-Benefit” OR “Cost-Utility Analysis” OR “Analyses, Cost-Utility” OR “Analysis, Cost-Utility” OR “Cost Utility Analysis” OR “Cost-Utility Analyses” OR “Economic Evaluation” OR “Economic Evaluations” OR “Evaluation, Economic” OR “Evaluations, Economic” OR “Marginal Analysis” OR “Analyses, Marginal” OR “Analysis, Marginal” OR “Marginal Analyses” OR “Cost Benefit” OR “Costs and Benefits” OR “Benefits and Costs” OR “Cost-Effectiveness Analysis” OR “Analysis, Cost-Effectiveness” OR “Cost Effectiveness Analysis” OR “incremental cost effectiveness ratio”

2. (“emerging country” [all fields] OR “emerging countries” [all fields] OR “emerging nation”[all fields] OR “emerging nations” [all fields] OR “emerging population”[all fields] OR “emerging populations”[all fields] OR "developing country"[tiab] OR "developing countries"[tiab] OR "developing nation"[tiab] OR "developing nations"[tiab] OR "developing population"[tiab] OR "developing populations"[tiab] OR "developing world"[tiab] OR "less developed country"[tiab] OR "less developed countries"[tiab] OR "less developed nation"[tiab] OR "less developed nations"[tiab] OR "less developed population"[tiab] OR "less developed populations"[tiab] OR "less developed world"[tiab] OR "lesser developed country"[tiab] OR "lesser developed countries"[tiab] OR "lesser developed nation"[tiab] OR "lesser developed nations"[tiab] OR "lesser developed population"[tiab] OR "lesser developed populations"[tiab] OR "lesser developed world"[tiab] OR "under developed country"[tiab] OR "under developed countries"[tiab] OR "under developed nation"[tiab] OR "under developed nations"[tiab] OR "under developed population"[tiab] OR "under developed populations"[tiab] OR "under developed world"[tiab] OR "underdeveloped country"[tiab] OR "underdeveloped countries"[tiab] OR "underdeveloped nation"[tiab] OR "underdeveloped nations"[tiab] OR "underdeveloped population"[tiab] OR "underdeveloped populations"[tiab] OR "underdeveloped world"[tiab] OR "middle income country"[tiab] OR "middle income countries"[tiab] OR "middle income nation"[tiab] OR "middle income nations"[tiab] OR "middle income population"[tiab] OR "middle income populations"[tiab] OR "low income country"[tiab] OR "low income countries"[tiab] OR "low income nation"[tiab] OR "low income nations"[tiab] OR "low income population"[tiab] OR "low income populations"[tiab] OR "lower income country"[tiab] OR "lower income countries"[tiab] OR "lower income nation"[tiab] OR "lower income nations"[tiab] OR "lower income population"[tiab] OR "lower income populations"[tiab] OR "underserved country"[tiab] OR "underserved countries"[tiab] OR "underserved nation"[tiab] OR "underserved nations"[tiab] OR "underserved population"[tiab] OR "underserved populations"[tiab] OR"underserved world"[tiab] OR "under served country"[tiab] OR "under served countries"[tiab] OR "under served nation"[tiab] OR "under served nations"[tiab] OR "under served population"[tiab] OR "under served populations"[tiab] OR "under served world"[tiab] OR "deprived country"[tiab] OR

"deprived countries"[tiab] OR "deprived nation"[tiab] OR "deprived nations"[tiab] OR "deprived

population"[tiab] OR "deprived populations"[tiab] OR "deprived world"[tiab] OR "poor country"[tiab]

OR "poor countries"[tiab] OR "poor nation"[tiab] OR "poor nations"[tiab] OR "poor population"[tiab] OR "poor populations"[tiab] OR "poor world"[tiab] OR "poorer country"[tiab] OR "poorer countries"[tiab] OR "poorer nation"[tiab] OR "poorer nations"[tiab] OR "poorer population"[tiab] OR "poorer populations"[tiab] OR "poorer world"[tiab] OR "developing economy"[tiab] OR "developing

economies"[tiab] OR "less developed economy"[tiab] OR "less developed economies"[tiab] OR "lesser developed economy"[tiab] OR "lesser developed economies"[tiab] OR "under developed economy"[tiab] OR "under developed economies"[tiab] OR "underdeveloped economy"[tiab] OR "underdeveloped economies"[tiab] OR "middle income economy"[tiab] OR "middle income economies"[tiab] OR "low income economy"[tiab] OR "low income economies"[tiab] OR "lower income economy"[tiab] OR "lower income economies"[tiab] OR "low gdp"[tiab] OR "low gnp"[tiab] OR "low gross domestic"[tiab] OR "low gross national"[tiab] OR "lower gdp"[tiab] OR "lower gnp"[tiab] OR "lower gross domestic"[tiab] OR "lower gross national"[tiab] OR lmic[tiab] OR lmics[tiab] OR "third world"[tiab] OR "lami country"[tiab] OR "lami countries"[tiab] OR "transitional country"[tiab] OR "transitional countries"[tiab] OR Africa[tiab]

OR Asia[tiab] OR Caribbean[tiab] OR West Indies[tiab] OR South America[tiab] OR Latin America[tiab] OR

Central America[tiab] OR "Atlantic Islands"[tiab] OR "Commonwealth of Independent States"[tiab] OR "Pacific Islands"[tiab] OR "Indian Ocean Islands"[tiab] OR "Eastern Europe"[tiab] OR Afghanistan[tiab] OR Albania[tiab] OR Algeria[tiab] OR Angola[tiab] OR Antigua[tiab] OR Barbuda[tiab] OR Argentina[tiab] OR Armenia[tiab] OR Armenian[tiab] OR Aruba[tiab] OR Azerbaijan[tiab] OR Bahrain[tiab] OR

Bangladesh[tiab] OR Barbados[tiab] OR Benin[tiab] OR Byelarus[tiab] OR Byelorussian[tiab] OR

Belarus[tiab] OR Belorussian[tiab] OR Belorussia[tiab] OR Belize[tiab] OR Bhutan[tiab] OR Bolivia[tiab]

OR Bosnia[tiab] OR Herzegovina[tiab] OR Hercegovina[tiab] OR Botswana[tiab] OR Brasil[tiab] OR

Brazil[tiab] OR Bulgaria[tiab] OR Burkina Faso[tiab] OR Burkina Fasso[tiab] OR Upper Volta[tiab] OR

Burundi[tiab] OR Urundi[tiab] OR Cambodia[tiab] OR Khmer Republic[tiab] OR Kampuchea[tiab] OR Cameroon[tiab] OR Cameroons[tiab] OR Cameron[tiab] OR Cape Verde[tiab] OR Central African Republic[tiab] OR Chad[tiab] OR Chile[tiab] OR China[tiab] OR Colombia[tiab] OR Comoros[tiab] OR Comoro Islands[tiab] OR Comores[tiab] OR Mayotte[tiab] OR Congo[tiab] OR Zaire[tiab] OR Costa Rica[tiab] OR Cote d'Ivoire[tiab] OR Ivory Coast[tiab] OR Croatia[tiab] OR Cuba[tiab] OR Cyprus[tiab] OR Czechoslovakia[tiab] OR "Czech Republic" [tiab] OR Slovakia[tiab] OR Slovak Republic[tiab] OR Djibouti[tiab] OR French Somaliland[tiab] OR Dominica[tiab] OR Dominican Republic[tiab] OR East Timor[tiab] OR East Timur[tiab] OR Timor Leste[tiab] OR Ecuador[tiab] OR Egypt[tiab] OR United Arab Republic[tiab] OR El Salvador[tiab] OR Eritrea[tiab] OR Estonia[tiab] OR Ethiopia[tiab] OR Fiji[tiab] OR Gabon[tiab] OR Gabonese Republic[tiab] OR Gambia[tiab] OR Gaza[tiab] OR Georgia Republic[tiab] OR Georgian Republic[tiab] OR Ghana[tiab] OR Gold Coast[tiab] OR Greece[tiab] OR Grenada[tiab] OR

Guatemala[tiab] OR Guinea[tiab] OR Guam[tiab] OR Guiana[tiab] OR Guyana[tiab] OR Haiti[tiab] OR

Honduras[tiab] OR Hungary[tiab] OR India[tiab] OR Maldives[tiab] OR Indonesia[tiab] OR Iran[tiab] OR Iraq[tiab] OR Jamaica[tiab] OR Jordan[tiab] OR Kazakhstan[tiab] OR Kazakh[tiab] OR Kenya[tiab] OR

Kiribati[tiab] OR Korea[tiab] OR Kosovo[tiab] OR Kyrgyzstan[tiab] OR Kirghizia[tiab] OR Kyrgyz Republic[tiab] OR Kirghiz[tiab] OR Kirgizstan[tiab] OR "Lao PDR"[tiab] OR Laos[tiab] OR Latvia[tiab] OR Lebanon[tiab] OR Lesotho[tiab] OR Basutoland[tiab] OR Liberia[tiab] OR Libya[tiab] OR Lithuania[tiab]OR

Macedonia[tiab] OR Madagascar[tiab] OR Malagasy Republic[tiab] OR Malaysia[tiab] OR Malaya[tiab] OR Malay[tiab] OR Sabah[tiab] OR Sarawak[tiab] OR Malawi[tiab] OR Nyasaland[tiab] OR Mali[tiab] OR Malta[tiab] OR Marshall Islands[tiab] OR Mauritania[tiab] OR Mauritius[tiab] OR Agalega Islands[tiab] OR

"Melanesia"[tiab] OR Mexico[tiab] OR Micronesia[tiab] OR Middle East[tiab] OR Moldova[tiab] OR Moldovia[tiab] OR Moldovian[tiab] OR Mongolia[tiab] OR Montenegro[tiab] OR Morocco[tiab] OR

Ifni[tiab] OR Mozambique[tiab] OR Myanmar[tiab] OR Myanma[tiab] OR Burma[tiab] OR Namibia[tiab] OR Nepal[tiab] OR Netherlands Antilles[tiab] OR New Caledonia[tiab] OR Nicaragua[tiab] OR Niger[tiab] OR Nigeria[tiab] OR Northern Mariana Islands[tiab] OR Oman[tiab] OR Muscat[tiab] OR Pakistan[tiab] OR Palau[tiab] OR Palestine[tiab] OR Panama[tiab] OR Paraguay[tiab] OR Peru[tiab] OR Philippines[tiab] OR Philipines[tiab] OR Phillipines[tiab] OR Phillippines[tiab] OR Poland[tiab] OR Portugal[tiab] OR Puerto Rico[tiab] OR Romania[tiab] OR Rumania[tiab] OR Roumania[tiab] OR Russia[tiab] OR Russian[tiab] OR

Rwanda[tiab] OR Ruanda[tiab] OR Saint Kitts[tiab] OR St Kitts[tiab] OR Nevis[tiab] OR Saint Lucia[tiab] OR St Lucia[tiab] OR Saint Vincent[tiab] OR St Vincent[tiab] OR Grenadines[tiab] OR Samoa[tiab] OR Samoan Islands[tiab] OR Navigator Island[tiab] OR Navigator Islands[tiab] OR Sao Tome[tiab] OR Saudi Arabia[tiab] OR Senegal[tiab] OR Serbia[tiab] OR Montenegro[tiab] OR Seychelles[tiab] OR Sierra Leone[tiab] OR Slovenia[tiab] OR Sri Lanka[tiab] OR Ceylon[tiab] OR Solomon Islands[tiab] OR

Somalia[tiab] OR Sudan[tiab] OR Suriname[tiab] OR Surinam[tiab] OR Swaziland[tiab] OR Syria[tiab] OR Syrian[tiab] OR Tajikistan[tiab] OR Tadzhikistan[tiab] OR Tadjikistan[tiab] OR Tadzhik[tiab] OR Tanzania[tiab] OR Thailand[tiab] OR Togo[tiab] OR Togolese Republic[tiab] OR Tonga[tiab] OR Trinidad[tiab] OR Tobago[tiab] OR Tunisia[tiab] OR Turkey[tiab] OR Turkmenistan[tiab] OR Turkmen[tiab] OR Tuvalu[tiab] OR Uganda[tiab] OR Ukraine[tiab] OR Uruguay[tiab] OR USSR[tiab] OR Soviet Union[tiab] OR Union of Soviet Socialist Republics[tiab] OR Uzbekistan[tiab] OR Uzbek OR Vanuatu[tiab] OR New Hebrides[tiab] OR Venezuela[tiab] OR Vietnam[tiab] OR Viet Nam[tiab] OR West Bank[tiab] OR Yemen[tiab] OR Yugoslavia[tiab] OR Zambia[tiab] OR Zimbabwe[tiab] OR Rhodesia[tiab] OR Developing Countries[Mesh] OR Africa[Mesh:NoExp] OR Africa, Northern[Mesh:NoExp] OR Africa South of the Sahara[Mesh:NoExp] OR Africa, Central[Mesh:NoExp] OR Africa, Eastern[Mesh:NoExp] OR Africa, Southern[Mesh:NoExp] OR Africa, Western[Mesh:NoExp] OR Asia[Mesh:NoExp] OR Asia,

Central[Mesh:NoExp] OR Asia, Southeastern[Mesh:NoExp] OR Asia, Western[Mesh:NoExp] OR Caribbean Region[Mesh:NoExp] OR West Indies[Mesh:NoExp] OR South America[Mesh:NoExp] OR Latin America[Mesh:NoExp] OR Central America[Mesh:NoExp] OR "Atlantic Islands"[Mesh:NoExp] OR "Commonwealth of Independent States"[Mesh:NoExp] OR "Pacific Islands"[Mesh:NoExp] OR "Indian Ocean Islands"[Mesh:NoExp] OR "Europe, Eastern"[Mesh:NoExp] OR Afghanistan[Mesh] OR Albania[Mesh] OR Algeria[Mesh] OR American Samoa[Mesh] OR Angola[Mesh] OR "Antigua and Barbuda"[Mesh] OR Argentina[Mesh] OR Armenia[Mesh] OR Azerbaijan[Mesh] OR Bahrain[Mesh] OR "Baltic States"[Mesh] OR Bangladesh[Mesh] OR Barbados[Mesh] OR Benin[Mesh] OR "Republic of Belarus"[Mesh] OR Belize[Mesh] OR Bhutan[Mesh] OR Bolivia[Mesh] OR Bosnia-Herzegovina[Mesh] OR Botswana[Mesh] OR Brazil[Mesh] OR Bulgaria[Mesh] OR Burkina Faso[Mesh] OR Burundi[Mesh] OR Cambodia[Mesh] OR Cameroon[Mesh] OR Cape Verde[Mesh] OR Central African Republic[Mesh] OR Chad[Mesh] OR Chile[Mesh] OR China[Mesh] OR Colombia[Mesh] OR Comoros[Mesh] OR Congo[Mesh] OR Costa Rica[Mesh] OR Cote d'Ivoire[Mesh] OR Croatia[Mesh] OR Cuba[Mesh] OR Cyprus[Mesh] OR Czechoslovakia[Mesh] OR Czech Republic[Mesh] OR Slovakia[Mesh] OR Djibouti[Mesh] OR "Democratic Republic of the Congo"[Mesh] OR "Democratic People's Republic of Korea"[Mesh] OR Dominica[Mesh] OR Dominican Republic[Mesh] OR East Timor[Mesh] OR Ecuador[Mesh] OR Egypt[Mesh] OR El Salvador[Mesh] OR Eritrea[Mesh] OR Estonia[Mesh] OR Ethiopia[Mesh] OR "Equatorial Guinea"[Mesh] OR Fiji[Mesh] OR "French Guiana"[Mesh] OR Gabon[Mesh] OR Gambia[Mesh] OR "Georgia (Republic)"[Mesh] OR Ghana[Mesh] OR Greece[Mesh] OR Grenada[Mesh] OR Guatemala[Mesh] OR Guinea[Mesh] OR Guinea-Bissau[Mesh] OR Guam[Mesh] OR Guyana[Mesh] OR Haiti[Mesh] OR Honduras[Mesh] OR Hungary[Mesh] OR "Independent State of Samoa"[Mesh] OR India[Mesh] OR Indonesia[Mesh] OR Iran[Mesh] OR Iraq[Mesh] OR Jamaica[Mesh] OR Jordan[Mesh] OR Kazakhstan[Mesh] OR Kenya[Mesh] OR Korea[Mesh] OR Kyrgyzstan[Mesh] OR Laos[Mesh] OR Latvia[Mesh] OR Lebanon[Mesh] OR Lesotho[Mesh] OR Liberia[Mesh] OR Libya[Mesh] OR Lithuania[Mesh] OR "Macedonia (Republic)"[Mesh] OR Madagascar[Mesh] OR Malawi[Mesh] OR Malaysia[Mesh] OR Mali[Mesh] OR Malta[Mesh] OR Mauritania[Mesh] OR Mauritius[Mesh] OR "Melanesia"[Mesh] OR Mexico[Mesh] OR Micronesia[Mesh] OR Middle East[Mesh:NoExp] ORMoldova[Mesh] OR Mongolia[Mesh] OR Montenegro[Mesh] OR Morocco[Mesh] OR Mozambique[Mesh] OR Myanmar[Mesh] OR Namibia[Mesh] OR Nepal[Mesh] OR Netherlands Antilles[Mesh] OR New Caledonia[Mesh] OR Nicaragua[Mesh] OR Niger[Mesh] OR Nigeria[Mesh] OR Oman[Mesh] OR Pakistan[Mesh] OR Palau[Mesh] OR Panama[Mesh] OR Papua New Guinea[Mesh] OR Paraguay[Mesh] OR Peru[Mesh] OR Philippines[Mesh] OR Poland[Mesh] OR Portugal[Mesh] OR Puerto Rico[Mesh] OR "Republic of Korea"[Mesh] OR Romania[Mesh] OR Russia[Mesh] OR "Russia (Pre-1917)"[Mesh] OR Rwanda[Mesh] OR "Saint Kitts and Nevis"[Mesh] OR Saint Lucia[Mesh] OR "Saint Vincent and the Grenadines"[Mesh] OR Samoa[Mesh] OR Saudi Arabia[Mesh] OR Senegal[Mesh] OR Serbia[Mesh] OR Montenegro[Mesh] OR Seychelles[Mesh] OR Sierra Leone[Mesh] OR Slovenia[Mesh] OR Sri Lanka[Mesh] OR Somalia[Mesh] OR South Africa[Mesh] OR Sudan[Mesh] OR Suriname[Mesh] OR Swaziland[Mesh] OR Syria[Mesh] OR Tajikistan[Mesh] OR Tanzania[Mesh] OR Thailand[Mesh] OR Togo[Mesh] OR Tonga[Mesh] OR "Trinidad and Tobago"[Mesh] OR Tunisia[Mesh] OR Turkey[Mesh] OR

Turkmenistan[Mesh] OR Uganda[Mesh] OR Ukraine[Mesh] OR Uruguay[Mesh] OR USSR[Mesh] OR Uzbekistan[Mesh] OR Vanuatu[Mesh] OR Venezuela[Mesh] OR Vietnam[Mesh] OR Yemen[Mesh] OR Yugoslavia[Mesh] OR Zambia[Mesh] OR Zimbabwe[Mesh] OR “Southern African Development Community” [all fields] OR “East African Community"[all fields] OR “West African Health Organisation"[all fields] OR “Sub Saharan Africa "[all fields] OR “SubSaharan Africa "[all fields])

3. “Mass Screenings” OR “Screening, Mass” OR “Screenings, Mass” OR “Screening” OR “Screenings” OR “screening test”

4. "diabetes mellitus"[MeSH Terms] OR "diabetes mellitus, type 2"[MeSH Terms] OR “Type 2 diabetes mellitus” OR T2DM OR "Diabetes mellitus" OR Diabetes OR DM OR “cardiovascular diseases” OR “cardiovascular diseases” [MeSH] OR CVD OR CVD [MeSH] OR “coronary artery disease” OR “coronary heart disease” OR stroke OR “cerebral Infarction” OR “intracerebral hemorrhage” OR "myocardial infarction" OR "brain infarction” OR “Cerebrovascular Disorders”[Majr:NoExp] OR “Stroke”[Mesh] OR “Thromboembolism”[Mesh] OR “Hemorrhage”[Mesh:NoExp] OR “Intracranial Hemorrhages”[Mesh] OR “Brain Ischemia”[Mesh] OR “Prothrombin Time”[Mesh] OR stroke OR strokes OR thromboembolism OR thromboembolisms OR thromboembolic OR thromboses OR hemorrhage OR hemorrhages OR hemorrhaging OR hemorrhagic OR haemorrhage OR haemorrhages OR haemorrhaging OR haemorrhagic OR ((“bleeding” OR bleed OR bleeds) AND (major OR risk OR event)) OR ((Systemic OR paradoxical OR crossed) AND (embolism OR embolisms)) OR ((brain OR cerebral OR brainstem OR “brain stem”) AND (ischemia OR ischaemia OR ischemias OR ischaemias OR infarction OR infarctions)) OR (transient AND (ischemic OR ischaemic OR ischaemia OR ischemia) AND (attack OR attacks)) OR TIA OR TIAs OR “cerebrovascular accident” OR “cerebrovascular accidents” OR CVA OR CVAs OR “brain vascular accident” OR “brain vascular accidents”

5. 1 AND 2 AND 3 AND 4

**Search outcome** (10.09.21): **564**

**Search strategy: Web of Science**

1. economic OR price OR pricing OR expenditure OR “value for money” OR budget* OR “return on investment” OR “value proposition” OR “Analyses, Cost-Benefit” OR “Cost Effectiveness” OR “Cost-Benefit Data” OR “Cost-Utility Analysis” OR “Economic Evaluation” OR “Marginal Analysis” OR “Cost Benefit” OR “Cost-Effectiveness Analysis” OR “incremental cost effectiveness ratio”

2. “emerging country” OR “developing country” OR “least developed countries” OR "less developed country" OR "lesser developed country" OR "under developed country" OR "middle income country" OR "low income country" OR "lower income country" OR “underserved country” OR “deprived country” OR “poor country” OR LMICs OR “Low and middle income countries” OR “Low income and middle income countries” OR Africa OR Asia OR Caribbean OR “West Indies” OR “South America” OR “Latin America” OR “Central America” OR "Atlantic Islands" OR "Commonwealth of Independent States" OR "Pacific Islands" OR "Indian Ocean Islands" OR "Eastern Europe"

3. “Mass Screenings” OR “Screening, Mass” OR “Screenings, Mass” OR “Screening” OR “Screenings” OR “screening test”

4. "diabetes mellitus" OR “Type 2 diabetes mellitus” OR T2DM OR Diabetes OR DM OR “cardiovascular diseases” OR “cardiovascular diseases” OR CVD OR “coronary artery disease” OR “coronary heart disease” OR stroke OR “cerebral Infarction” OR “intracerebral hemorrhage” OR "myocardial infarction" OR "brain infarction” OR “Cerebrovascular Disorders” OR “Thromboembolism” OR “Hemorrhage” OR “Intracranial Hemorrhages” OR “Brain Ischemia” OR “Prothrombin Time” OR thromboembolism OR thromboembolisms OR thromboembolic OR thromboses OR hemorrhage OR “bleeding” OR bleed OR Systemic OR paradoxical OR crossed OR embolism OR brain OR cerebral OR brainstem OR ischemia OR infarction OR transient OR ischemic OR TIA OR TIAs OR “cerebrovascular accident” OR “cerebrovascular accidents” OR CVA OR CVAs OR “brain vascular accident” OR “brain vascular accidents”

5. 1 AND 2 AND 3 AND 4

Filter: All fields

**Search outcome** (10.09.21): **387**

**Search strategy: SCOPUS**

1. economic OR price OR pricing OR expenditure OR “value for money” OR budget OR “return on investment” OR “value proposition” OR “Analyses, Cost-Benefit” OR “Cost Effectiveness” OR “Cost-Benefit Data” OR “Cost-Utility Analysis” OR “Economic Evaluation” OR “Marginal Analysis” OR “Cost Benefit” OR “Cost-Effectiveness Analysis” OR “incremental cost effectiveness ratio”

2. “emerging country” OR “developing country” OR “least developed countries” OR "less developed country" OR "lesser developed country" OR "under developed country" OR "middle income country" OR "low income country" OR "lower income country" OR “underserved country” OR “deprived country” OR “poor country” OR LMICs OR “Low and middle income countries” OR “Low income and middle income countries” OR Africa OR Asia OR Caribbean OR “West Indies” OR “South America” OR “Latin America” OR “Central America” OR "Atlantic Islands" OR "Commonwealth of Independent States" OR "Pacific Islands" OR "Indian Ocean Islands" OR "Eastern Europe"

3. “Mass Screenings” OR “Screening, Mass” OR “Screenings, Mass” OR “Screening” OR “Screenings” OR “screening test”

4. “diabetes mellitus” OR “Type 2 diabetes mellitus” OR T2DM OR Diabetes OR DM OR “cardiovascular diseases” OR CVD OR “coronary artery disease” OR “coronary heart disease” OR stroke OR “cerebral Infarction” OR “intracerebral hemorrhage” OR “myocardial infarction” OR “brain infarction” OR “Cerebrovascular Disorders” OR Thromboembolism OR Hemorrhage OR “Intracranial Hemorrhages” OR “Brain Ischemia” OR “Prothrombin Time” OR thromboembolism OR thromboembolisms OR thromboembolic OR thromboses OR hemorrhage OR bleeding OR bleed OR Systemic OR paradoxical OR crossed OR embolism OR brain OR cerebral OR brainstem OR ischemia OR infarction OR transient OR ischemic OR TIA OR TIAs OR “cerebrovascular accident” OR “cerebrovascular accidents” OR CVA OR CVAs OR “brain vascular accident” OR “brain vascular accidents”

5. 1 AND 2 AND 3 AND 4

Filter: Title, Abstract, Keywords

**Search outcome** (10.09.21): **466**

**EconLit**

1. economic OR price OR pricing OR expenditure OR "value for money" OR budget OR "return on investment" OR "value proposition" OR "Analyses, Cost-Benefit" OR "Cost Effectiveness" OR "Cost-Benefit Data" OR "Cost-Utility Analysis" OR "Economic Evaluation" OR "Marginal Analysis" OR "Cost Benefit" OR "Cost-Effectiveness Analysis" OR "incremental cost effectiveness ratio"

2. "emerging country" OR "developing country" OR "least developed countries" OR "less developed country" OR "lesser developed country" OR "under developed country" OR "middle income country" OR "low income country" OR "lower income country" OR "undeserved country" OR "deprived country" OR "poor country" OR laics OR "Low and middle income countries" OR "Low income and middle income countries" OR Africa OR Asia OR Caribbean OR "West Indies" OR "South America" OR "Latin America" OR "Central America" OR "Atlantic Islands" OR "Commonwealth of Independent States" OR "Pacific Islands" OR "Indian Ocean Islands" OR "Eastern Europe"

3. "Mass Screenings" OR "Screening, Mass" OR "Screenings, Mass" OR "Screening" OR "Screenings" OR "screening test"

4. "diabetes mellitus" OR "Type 2 diabetes mellitus" OR T2DM OR Diabetes OR DM OR "cardiovascular diseases" OR CVD OR "coronary artery disease" OR "coronary heart disease" OR stroke OR "cerebral Infarction" OR "intracerebral hemorrhage" OR "myocardial infarction" OR "brain infarction" OR "Cerebrovascular Disorders" OR Thromboembolism OR Hemorrhage OR "Intracranial Hemorrhages" OR "Brain Ischemia" OR "Prothrombin Time" OR thromboembolism OR thromboembolism OR thromboembolic OR thromboses OR hemorrhage OR breeding OR bleed OR Systemic OR paradoxical OR crossed OR embolism OR brain OR cerebral OR brainstem OR ischemia OR infarction OR transient OR ischemic OR TIA OR ties OR "cerebrovascular accident" OR "cerebrovascular accidents" OR CVA OR CVAs OR "brain vascular accident" OR "brain vascular accidents"

Filter: All fields

**Search outcome** (13.09.21): **12**

**EMBASE (OVID)**

1. economic OR price OR pricing OR expenditure OR "value for money" OR budget OR "return on investment" OR "value proposition" OR "Analyses, Cost-Benefit" OR "Cost Effectiveness" OR "Cost-Benefit Data" OR "Cost-Utility Analysis" OR "Economic Evaluation" OR "Marginal Analysis" OR "Cost Benefit" OR "Cost-Effectiveness Analysis" OR "incremental cost effectiveness ratio"

2. "emerging country" OR "developing country" OR "least developed countries" OR "less developed country" OR "lesser developed country" OR "under developed country" OR "middle income country" OR "low income country" OR "lower income country" OR "undeserved country" OR "deprived country" OR "poor country" OR laics OR "Low and middle income countries" OR "Low income and middle income countries" OR Africa OR Asia OR Caribbean OR "West Indies" OR "South America" OR "Latin America" OR "Central America" OR "Atlantic Islands" OR "Commonwealth of Independent States" OR "Pacific Islands" OR "Indian Ocean Islands" OR "Eastern Europe"

3. "Mass Screenings" OR "Screening, Mass" OR "Screenings, Mass" OR "Screening" OR "Screenings" OR "screening test"

4. "diabetes mellitus" OR "Type 2 diabetes mellitus" OR T2DM OR Diabetes OR DM OR "cardiovascular diseases" OR CVD OR "coronary artery disease" OR "coronary heart disease" OR stroke OR "cerebral Infarction" OR "intracerebral hemorrhage" OR "myocardial infarction" OR "brain infarction" OR "Cerebrovascular Disorders" OR Thromboembolism OR Hemorrhage OR "Intracranial Hemorrhages" OR "Brain Ischemia" OR "Prothrombin Time" OR thromboembolism OR thromboembolism OR thromboembolic OR thromboses OR hemorrhage OR breeding OR bleed OR Systemic OR paradoxical OR crossed OR embolism OR brain OR cerebral OR brainstem OR ischemia OR infarction OR transient OR ischemic OR TIA OR ties OR "cerebrovascular accident" OR "cerebrovascular accidents" OR CVA OR CVAs OR "brain vascular accident" OR "brain vascular accidents"

Filter: All fields

**Search outcome** (13.09.21): **755**
